# A few essential genetic loci distinguish *Penstemon* species with flowers adapted to pollination by bees or hummingbirds

**DOI:** 10.1371/journal.pbio.3002294

**Published:** 2023-09-28

**Authors:** Carolyn A. Wessinger, Amanda M. Katzer, Paul M. Hime, Mark D. Rausher, John K. Kelly, Lena C. Hileman

**Affiliations:** 1 Department of Biological Sciences, University of South Carolina, Columbia, South Carolina, United States of America; 2 Department of Ecology and Evolutionary Biology, University of Kansas, Lawrence, Kansas, United States of America; 3 Biodiversity Institute and Natural History Museum, University of Kansas, Lawrence, Kansas, United States of America; 4 Department of Biology, Duke University, Durham, North Carolina, United States of America; Institute of Science and Technology Austria (IST Austria), AUSTRIA

## Abstract

In the formation of species, adaptation by natural selection generates distinct combinations of traits that function well together. The maintenance of adaptive trait combinations in the face of gene flow depends on the strength and nature of selection acting on the underlying genetic loci. Floral pollination syndromes exemplify the evolution of trait combinations adaptive for particular pollinators. The North American wildflower genus *Penstemon* displays remarkable floral syndrome convergence, with at least 20 separate lineages that have evolved from ancestral bee pollination syndrome (wide blue-purple flowers that present a landing platform for bees and small amounts of nectar) to hummingbird pollination syndrome (bright red narrowly tubular flowers offering copious nectar). Related taxa that differ in floral syndrome offer an attractive opportunity to examine the genomic basis of complex trait divergence. In this study, we characterized genomic divergence among 229 individuals from a *Penstemon* species complex that includes both bee and hummingbird floral syndromes. Field plants are easily classified into species based on phenotypic differences and hybrids displaying intermediate floral syndromes are rare. Despite unambiguous phenotypic differences, genome-wide differentiation between species is minimal. Hummingbird-adapted populations are more genetically similar to nearby bee-adapted populations than to geographically distant hummingbird-adapted populations, in terms of genome-wide *d*_*XY*_. However, a small number of genetic loci are strongly differentiated between species. These approximately 20 “species-diagnostic loci,” which appear to have nearly fixed differences between pollination syndromes, are sprinkled throughout the genome in high recombination regions. Several map closely to previously established floral trait quantitative trait loci (QTLs). The striking difference between the diagnostic loci and the genome as whole suggests strong selection to maintain distinct combinations of traits, but with sufficient gene flow to homogenize the genomic background. A surprisingly small number of alleles confer phenotypic differences that form the basis of species identity in this species complex.

## Abstract 2

A genomic study of 229 individuals from a Penstemon species complex that includes both bee and hummingbird pollination syndromes reveals that although genome-wide differentiation between species is minimal, about 20 "species-diagnostic loci," with fixed differences between syndromes, are sprinkled throughout the genome in high recombination regions.

## Introduction

Many adaptations are complex, with multiple component traits contributing to a shared function. Although the origin and evolution of complex adaptations historically posed a challenge (e.g., [1,2]), recent theoretical and empirical studies have identified population genetic mechanisms and predicted patterns for the evolution of complex adaptations (reviewed by [3–5]). Local adaptation to a new environment can spur evolutionary change favoring a new trait combination (e.g., [6–8]), involving genetic substitution at multiple loci that affect the different component traits. Mutations may be individually favored by selection or may experience correlated selection due to epistatic effects among traits on fitness. Regardless of the nature of selection acting on individual mutations, a fundamental problem for adaptation in suites of traits is that recombination tends to break apart the favorable multi-locus combinations of alleles. Therefore, divergence during local adaptation is vulnerable to the effects of gene flow that can scramble or swamp out associations between alleles [9,10].

Local adaptation in the face of gene flow thus requires strong selection to maintain multi-trait adaptive phenotypes. Linkage disequilibrium between relevant loci can be maintained through strong selection on individual traits or combinations of traits coupled with nonrandom mating [11,12]. In addition, the effect sizes of adaptive loci and their relative positions in the genome (the genetic architecture of the traits) are expected to affect the outcome of local adaptation with gene flow [5,13]. A “concentrated architecture” with close linkage of loci or recombination suppression by inversions can counteract the diluting effects of recombination [13–18]. In cases where closely related taxa exhibit differences in complex traits, population genomic data is well-suited to describe the opposing effects of selection and gene flow on loci determining trait combinations. An expected pattern is that adaptive loci will show genetic differentiation between locally adapting populations, with less differentiation across the rest of the genome due to gene flow [13,19,20].

Floral syndromes are quintessential complex adaptations made up of morphological traits, nectar traits, flower color, and scent—where specific combinations of traits reflect adaptation to a particular type of pollinator or abiotic agent [21,22]. Adaptive transitions in floral syndrome can occur rapidly, leading to closely related species in the same geographic area that differ in floral syndrome [23–26]. The North American perennial wildflower genus *Penstemon* has experienced repeated shifts from bee to hummingbird pollination syndrome. Most of the roughly 290 *Penstemon* species are adapted to bee pollination, usually exhibiting wide blue or purple flowers with a landing platform formed by lower petal lobes and that produce small amounts of nectar, whereas hummingbird-adapted *Penstemon* flowers are bright red, narrowly tubular, lack the landing platform, have elongated stamen filaments and styles, and produce copious nectar [27]. These alternate floral syndromes show clear separation in multi-trait space corresponding to alternate peaks on a multi-trait adaptive landscape [27,28]. Upwards of 20 repeated origins of hummingbird adaptation are distributed widely across independent *Penstemon* lineages, and hummingbird-adapted species are usually sister to bee-adapted species [29,30], indicating numerous recent origins of hummingbird syndrome. This remarkable evolutionary replication makes *Penstemon* an attractive system for studying the genomic basis of rapid complex trait divergence, the maintenance of divergent complex adaptations in the face of gene flow, and the resulting influence on genome-wide patterns of genetic differentiation.

Here, we investigated patterns of genetic divergence within a focal complex of 3 species that includes the hummingbird-adapted species *P*. *barbatus* and 2 bee-adapted species that are phenotypically and genetically similar: *P*. *neomexicanus* and *P*. *virgatus*. These species are closely related [30,31] and have partially overlapping distributions at mid to high elevations in the southern Rocky Mountains and sky islands of Arizona and New Mexico. In locations where bee- and hummingbird-adapted species occur sympatrically, obvious interspecific hybrids are rare. We present population genomic data—from populations sampled widely across species’ ranges—that indicates a surprisingly small number of unlinked loci found throughout the genome specify the evolutionarily derived identity of *P*. *barbatus* and maintain the corresponding pollination syndrome divergence. By contrast, most genome-wide variation does not associate with species identity, and appears as if collected from a single well-mixed species, indicating these species share substantial amounts of allelic diversity. Our results are consistent with selection maintaining multi-locus genotypes that determine pollination syndromes despite the homogenizing effects of gene flow.

## Results

### Hummingbird-adapted *P*. *barbatus* is genetically intertwined with related bee-adapted species despite floral syndrome divergence

To understand patterns of genetic differentiation within the focal species complex, we first assembled a reference genome for *P*. *barbatus*. We obtained pseudochromosomes using a combination of sequencing approaches followed by alignment to a genetic linkage map to help order and orient scaffolds (S1 Text). Next, we performed multiplexed shotgun genotyping (MSG) [32] to generate shallow population genomic data for 15 populations of *P*. *barbatus*, 3 populations of *P*. *neomexicanus*, and 8 populations of *P*. *virgatus* that are widely distributed throughout Arizona, Colorado, and New Mexico (S1 Table). We sampled approximately 8 individuals per population (see Methods). Mapping the MSG data to the genome assembly, we scored field-sampled individuals at 168 million sites in the genome (approximately 28% of the 599 Mbp reference genome length). Accounting for missing data across individuals, the average depth per site was 0.57×.

**Fig 1:**
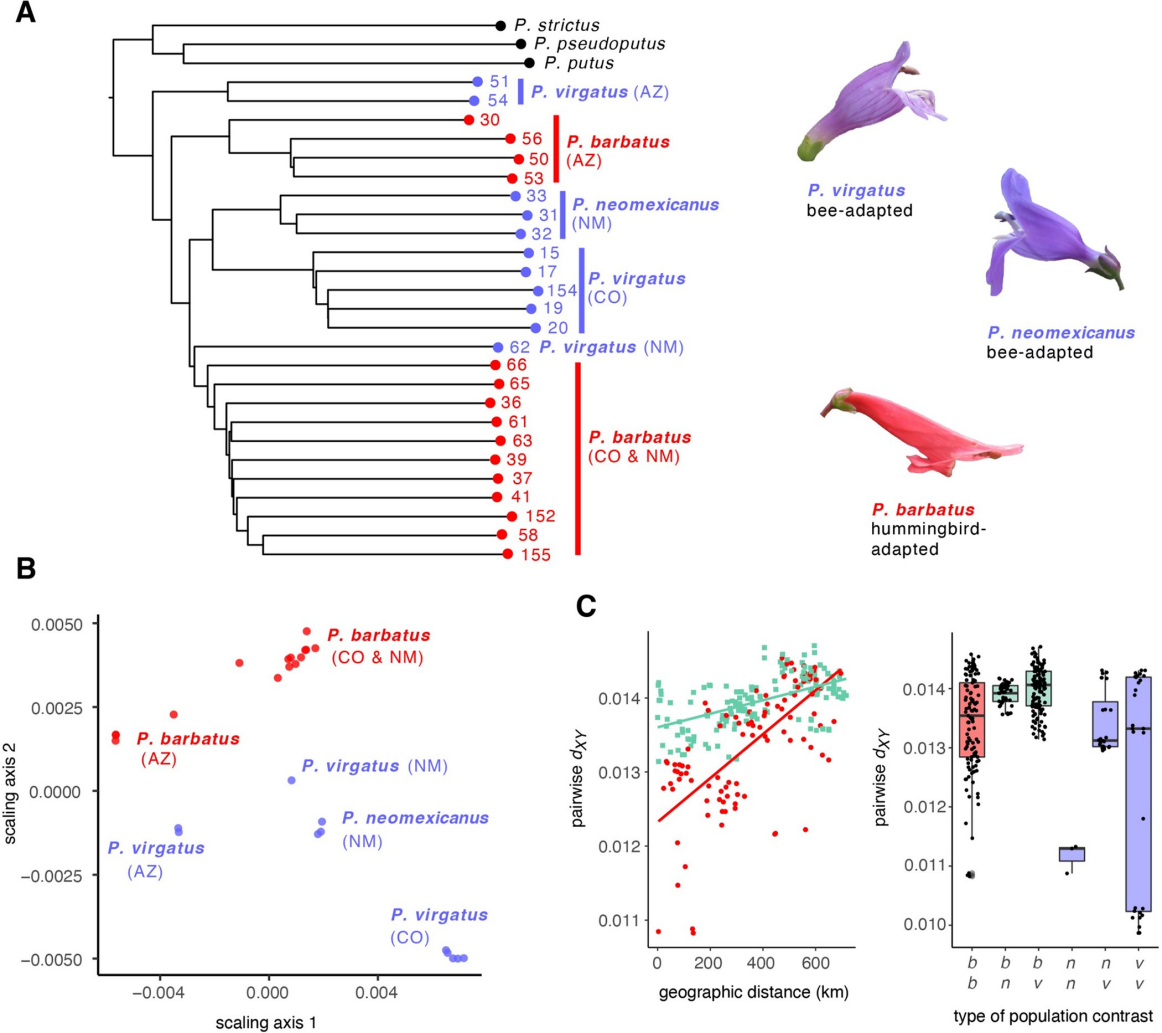
Genetic relationships between sampled *P*. *barbatus*, *P*. *neomexicanus*, and *P*. *virgatus* populations based a matrix of average pairwise genetic distance between populations. (A) Neighbor-joining tree. (B) Multidimensional scaling applied to the matrix. (C) Average pairwise genetic distance (*d*_*XY*_) between populations as a function of geographic distance and summarized by contrast type. Red: pairwise contrasts between *P*. *barbatus* populations, green: pairwise contrasts between *P*. *barbatus* vs. bee-adapted populations. AZ: Arizona, CO: Colorado, NM: New Mexico, *b*: *P*. *barbatus*, *n*: *P*. *neomexicanus*, *v*: *P*. *virgatus*. Photographs by C.A.W. The data underlying this figure can be found in 10.5061/dryad.xpnvx0kmp.

From our MSG data, we calculated genome-wide average pairwise genetic distance (*d*_*XY*_) between populations. The pairwise genetic distance matrix is depicted as a neighbor-joining tree (in Fig 1A) and as a multidimensional scaling plot (in Fig 1B), but both reveal the same intriguing pattern: eastern hummingbird-adapted *P*. *barbatus* populations from Colorado and New Mexico are more closely related to eastern populations of the bee-adapted species *P*. *neomexicanus* and *P*. *virgatus* than they are to western populations of *P*. *barbatus* from northern Arizona. Median *d*_*XY*_ between sampled *P*. *barbatus* populations is 0.0143 and median *d*_*XY*_ between sampled populations that differ in floral syndrome is 0.0163, with substantial overlap in the degree of divergence between these 2 categories (Fig 1C and S2 Table). Genetic distance between pairs of populations increases with geographic distance between them (Fig 1C), indicating that nearby populations are connected by gene flow. Note that this pattern of isolation-by-distance is recovered for interspecific population pairs, suggesting genetic exchange between local bee- and hummingbird-adapted populations. The same pattern is found if genetic differentiation is measured by *F*_*ST*_ between populations (see S1 Fig and S2 Table). Yet, despite genetic intermingling of these species, at a local spatial scale, pairs of *P*. *barbatus* populations are less divergent than interspecific population pairs (Fig 1C).

### 21 Regions distributed throughout the genome diagnose species differences

Genome-wide patterns (Fig 1) suggest substantial genetic mixing between species despite their strong differentiation in floral traits and in their pollinators. We next sought to identify outlier single nucleotide polymorphisms (SNPs) that distinguish *P*. *barbatus* from its bee-adapted relatives. We conducted an SNP-by-SNP association scan using likelihood-based models that properly accommodate genotype uncertainty inherent to our shallow sequencing data. At each SNP, we compared the likelihood of 2 models: (H_1_) genotype frequencies are allowed to differ between *P*. *barbatus* and bee-adapted species, and (H_0_) frequencies are the same in *P*. *barbatus* versus bee-adapted species. We used a likelihood ratio test (LRT) to compare the 2 models. This approach identifies SNPs predictive of “species identity,” the phenotypic differences that are recognized to form the basis of species identification in practice, including pollination syndrome traits. The LRT statistic increases with the magnitude of allele frequency divergence and with increasing statistical confidence in the estimate for divergence (S2 Fig).

**Fig 2:**
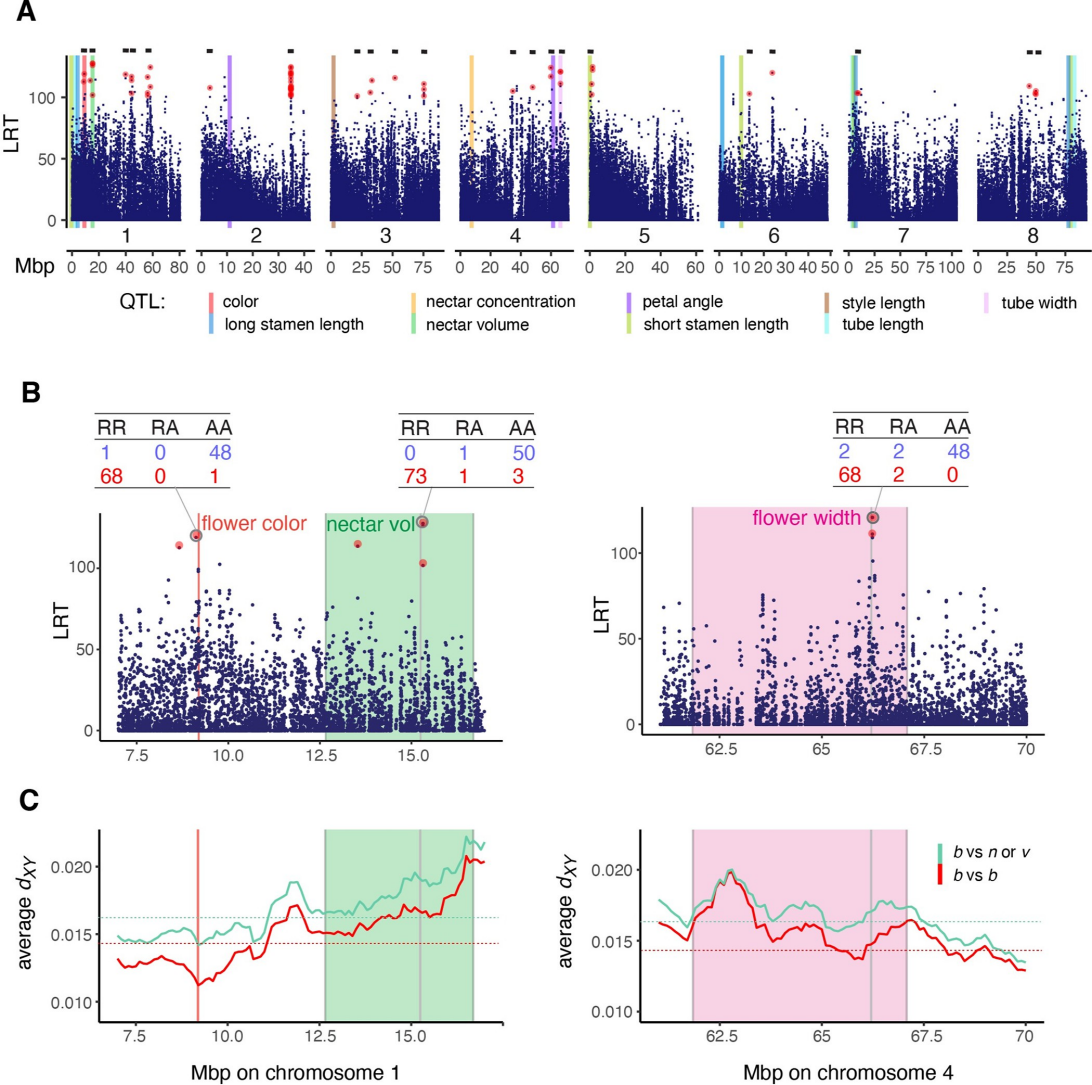
SNP genotype associations with pollination syndrome, plotted according to the magnitude of the LRT statistic. Points highlighted in red are outlier SNPs (difference in allele frequency > 0.9 and LRT > 100). (A) Genome-wide view, horizontal black bars at top of each panel indicate the 21 regions with highly differentiated SNPs, vertical colored lines indicate peak LOD positions for floral trait QTLs identified in a genetic cross between floral syndromes. (B) Insets show SNPs of interest near major QTLs, with colored shaded areas representing 1.5-LOD confidence intervals of QTLs for nectar volume and flower width. Circled SNPs correspond to SNPs cosegregating most strongly with floral syndrome. Tables show genotype counts in bee-adapted (blue) vs. hummingbird-adapted (red) individuals for circled SNPs. (C) Average *d*_*XY*_ in the vicinity of major floral trait QTLs, plotted in moving averages of ten 100-kb windows. Red trace: average pairwise *d*_*XY*_ between *P*. *barbatus* populations, green trace: average pairwise *d*_*XY*_ between *P*. *barbatus* vs. bee-adapted populations. Dotted lines indicate genome-wide average values. *b*: *P*. *barbatus*, *n*: *P*. *neomexicanus*, *v*: *P*. *virgatus*. The data underlying this figure can be found in 10.5061/dryad.xpnvx0kmp. LOD, logarithm of odds; LRT, likelihood ratio test; QTL, quantitative trait locus; SNP, single nucleotide polymorphism.

Most SNPs do not predict species identity (S2 Fig), but a small subset are strongly associated with species identity and are distributed throughout the genome (Fig 2). We identified a set of 63 SNPs that had LRT statistic values greater than 100 (corresponding to a genome-wide Bonferroni-corrected *p* < 10^−10^) and had differences in allele frequencies between pollination syndromes greater than 0.9. These represent outlier SNPs that show nearly fixed differences between pollination syndromes with high confidence (S2 Fig). We collapsed outlier SNPs within 2 Mbp of each other, finding a set of 21 species-diagnostic genomic regions (horizontal black bars in Fig 2), each of which harbors SNPs that are nearly fixed differences between *P*. *barbatus* versus the bee-adapted species (Fig 2 and S3 Table). We note that different thresholds could be used to assign SNPs to outlier status or to bin outlier SNPs to species-diagnostic regions that would return a different number of SNPs and species-diagnostic regions. Regardless of the exact number of loci, our observation is that only a small number of SNPs are in strong association with species identity—the majority of the genome is not associated with species. These species-diagnostic SNPs are in linkage disequilibrium with each other, because each is strongly associated with species identity, but not with neighboring SNPs that have low association with species identity.

### Multiple species-diagnostic loci correspond to previously mapped floral trait QTLs

To determine if species-diagnostic loci might be responsible for the floral syndrome differences between species, we related the genomic locations of diagnostic SNPs (Fig 2) to those of known floral trait quantitative trait loci (QTLs). Previously, we mapped floral syndrome QTLs using a genetic cross between *P*. *barbatus* and *P*. *neomexicanus* [33]. Because our new genome assembly and linkage map now allow us to identify the physical locations of these QTLs, we reestimated floral trait QTLs using genomic and phenotypic data from 133 F2 individuals and determined their physical location within the genome assembly based on their genetic position (see Materials and methods).

Consistent with Wessinger and colleagues [33], we find that 3 key traits defining pollination syndrome in *Penstemon*—flower color, nectar volume, and flower width—involve single QTLs of major effect (S3 Fig and S4 Table). Flower color variation maps to a single location on chromosome 1 that contains the anthocyanin pathway gene *Flavonoid 3′*,*5′-hydroxylase*. Loss-of-function mutations in this gene cause flower color variation in this system [34]. A single major effect QTL for nectar volume also maps to chromosome 1, approximately 10.5 cM away from the flower color gene. Flower width also involves a single major effect QTL that maps to chromosome 4. Other aspects of floral morphology are polygenic; these traits include flower length, stamen filament length, style length, and the angle of the lower petal.

We find a species-diagnostic locus in the immediate vicinity (within 100 kb of the peak position) of each of the 3 major-effect QTLs, including 8 outlier SNPs that fall within 100 kb of peak logarithm of odds (LOD) positions of QTLs: 1 at the color locus, 4 within the nectar volume QTL, and 3 within the width QTL (S3 Table). To determine whether this overlap of outlier SNPs and QTL peak positions is greater than expected by chance, we randomly permuted the genomic positions of the SNP dataset 10,000 times and tallied the number of SNPs that fell within 100 kb of a QTL peak position. The mean number of SNPs meeting this threshold in permutations was 0.80 and the maximum number was 5, making the observed value of 8 SNPs well outside the expectation under random chance (S4 Fig). An addition 2 species-diagnostic regions occurred within 2 Mbp of QTL peaks for stamen length (chromosome 5) and petal angle (chromosome 4) (Figs 2 and S3 and S3 Table). All 5 species-diagnostic regions mentioned here are within 1.5 peak LOD confidence intervals of their associated floral trait QTL (S4 Table).

### Genomic differentiation between hummingbird- and bee-adapted taxa is highly localized

We examined the genomic landscape of divergence between the floral syndromes to determine whether identified outlier SNPs—including those near key floral traits—are located within extended genomic islands of elevated divergence. Specifically, we examined levels of within-population polymorphism (π) and between-population *d*_*XY*_ for 500 kb and 100 kb non-overlapping genomic windows. Background levels of π and *d*_*XY*_ are expected to be shaped by intrinsic genomic features such as local gene density and recombination rate. Indeed, gene density and recombination rate both vary substantially across the *P*. *barbatus* genome (S5 Fig). Gene-poor regions of the genome tend to experience low recombination (and may be centromeric), whereas high recombination regions tend to be gene-rich (S5 and S6 Figs). Across genomic windows, within-population polymorphism (π) shows a negative relationship with the fraction of coding sites and a positive relationship with recombination rate (S6 Fig). Levels of *d*_*XY*_ between *P*. *barbatus* populations and between *P*. *barbatus* versus bee-adapted species are strongly positively correlated with levels of within-population π (S5 and S6 Figs), suggesting that the landscape of divergence between populations and between species largely matches genome-wide patterns of within-population polymorphism.

Floral syndrome QTLs are dispersed throughout the genome in regions that experience relatively high rates of recombination (S5 and S6 Figs). Notably, levels of *d*_*XY*_ between populations that differ in floral syndrome are not strongly elevated above the genome-wide pattern in 100 kb genomic windows that overlap the floral QTLs (Figs 2 and S6), indicating a lack of extended elevated genomic differentiation surrounding species-diagnostic loci. Results are similar for *F*_*ST*_ (S6 Fig). Thus, although species-diagnostic regions were often found near mapped floral QTLs, these regions on the whole were not exceptionally differentiated at the scale of a genomic window. These results suggest fine-scale species differentiation across the genome, much finer than the scale by which we resolved QTL in our mapping study.

## Discussion

### Complex multi-trait differentiation in the face of gene flow

Our genomic data suggest that the hummingbird-adapted *P*. *barbatus* and its bee-adapted relatives, *P*. *virgatus* and *P*. *neomexicanus*, form a species complex with porous boundaries between taxa. Members of this complex are delimited based on divergent morphological traits and the absence of intermediate combinations of floral traits that inevitably result from interspecific hybridization. Indeed, only occasionally are hybrids between *P*. *barbatus* and *P*. *virgatus* or *P*. *neomexicanus* noted within natural populations [C.W. pers. obs., 35]. Yet, despite their strongly differentiated floral traits and distinct pollinators, the species show genetic intermingling at a regional scale. We found a positive relationship between pairwise genetic distance and geographic distance for both intra- and interspecific population contrasts. This suggests genetic exchange between nearby populations even if they differ in pollination syndrome. Together, these observations are consistent with complex floral differentiation in the face of gene flow. Several other studies have examined genetic divergence between partially sympatric species that differ in floral syndrome and primary pollinator. These study systems also find that floral trait differentiation exceeds overall levels of genetic differentiation in genome-wide marker data. Examples include related *Ipomopsis* species [36], *Aquilegia* species [37–39], *Mimulus aurantiacus* pollinator ecotypes [40], and *Ipomoea* species [41]. Thus, divergent natural selection may commonly maintain floral syndrome differences despite gene flow. An alternative explanation for the substantial shared genetic variation is that speciation may have occurred so recently that there has not yet been sufficient time for genetic drift to have sorted variation within lineages. While it is likely true that divergence occurred very recently and some shared variation may still be sorting, this view fails to explain the pattern of genetic isolation-by-distance for interspecific population contrasts.

Are floral syndrome hybrids rare because they are rarely made (syndromes rarely cross) or because hybrids are rapidly eliminated by selection? In several classic systems (e.g., *Mimulus* [42–44], *Iris* [45,46], *Penstemon* [47], *Ipomopsis* [48], *Petunia* [49], and *Aquilegia* [50]), it has been shown that hybrids are rare because mating is highly assortative due to contrasting preferences of different pollinators for different floral syndromes [23,25,26,51]. By contrast, few studies have tested whether hybrids between species with different pollinator syndromes have reduced fitness in the wild. Studies in *Mimulus*, *Iris*, *Nicotiana*, *Ipomopsis*, and other species of *Penstemon* have found that floral syndrome hybrids (e.g., F1 or F2 hybrids) often are visited by pollinators at rates that are intermediate between rates for the 2 parent species, leading to similar total pollination rates [44,47,52–54]. Our genomic results (Fig 2) show little genome-wide differentiation between nearby bee- and hummingbird-adapted populations, which implies hybridization occurs. Perhaps F1 hybrids are occasionally produced between bee- and hummingbird-pollinated *Penstemon* and a subset of their progeny backcross to one or both of the parental species. This partial success of hybrids is required to homogenize genetic backgrounds between species, even as selection eliminates hybrid genotypes at loci determining syndrome. Unlike other study systems (e.g., [37,54–56]), clear hybrid zones that serve as a bridge for gene flow have not been found in the *Penstemon* species complex described here. This absence is notable given our geographically broad field sampling in this system. However, even the rare formation of hybrids with lower relative fitness can bridge species through gene flow [57]. Therefore, we conclude that very rare hybridization events between hummingbird-adapted *P*. *barbatus* and its bee-adapted relatives must be enough to generate the observed genetic intermingling between species.

### Only a few regions of the genome diagnose species identity and floral differences

Our SNP-by-SNP likelihood-based genome scan successfully detected SNPs that diagnose the species, some of which appear linked to alleles underlying pollinator adaptation. We found relatively few species-diagnostic loci in the genome—most of the genome-wide variation showed little association with species identity. SNP outliers are found within 100 kb of LOD positions for the major effect QTLs for flower color, nectar production, and flower width that critically define floral syndrome differences in this system. The fact that these outliers co-occur with floral trait QTLs validates our likelihood-based approach, as it confirms that the SNP associations are identifying key QTLs that distinguish the species. We have greater confidence in the QTL peak positions for major-effect QTLs compared with those for polygenic traits because our current QTL analysis is underpowered for detecting and resolving the positions of smaller-effect QTLs. Yet reassuringly, we see outliers within 2 Mbp of QTL peak LOD positions for the polygenic traits stamen length and petal angle. The outlier regions that do not overlap with floral trait QTLs could represent smaller effect QTLs that are difficult to precisely map using our small QTL mapping population, or they might represent unmeasured floral, vegetative, or life history phenotypes that distinguish *P*. *barbatus* from its bee-adapted relatives. An expanded QTL study in the system, with more traits scored and more individuals in the mapping population, will improve our understanding of the genetic architecture and physical location of QTLs that confer phenotypic differences.

A limitation of our population genomic data is that MSG-based genotype calls are fragmentary and only cover approximately 28% of the genome. We are unable to identify functional effects of underlying species-diagnostic loci with the current data and there may be additional species-diagnostic regions not covered by our dataset. Our study might therefore underestimate the total number of divergent loci. The absolute number of divergent loci is perhaps less compelling than the striking difference between variation at a small number of species-diagnostic loci and variation in the rest of the genome that shows low association with species identity. Future genome-wide association scans in this system using whole genome sequencing will increase our ability to identify SNP associations at a fine scale, providing substantially greater resolution than QTL mapping, and point towards candidate genes that determine range-wide species differences.

A concentrated genetic architecture (e.g., trait differences controlled by few loci with large effect, extensive pleiotropy involving many traits, or tight linkage between loci associated with different traits) in theory would allow the integrity of a complex trait to be maintained despite hybridization [5,18]. Several studies that have investigated the genetic architecture of floral syndrome divergence identify at least a few loci of major effect [58–65] and colocalization of QTLs affecting different traits due to tight linkage or pleiotropy [38,40,64–68]. In the *P*. *barbatus* x *P*. *neomexicanus* genetic cross, 3 key traits that define pollination syndrome involve major effect QTLs—flower color, nectar volume, and flower width. Moreover, QTLs for flower length and stamen filament length colocalize, perhaps reflecting pleiotropic function to increase floral tissue length acting in multiple floral whorls [33]. These characteristics thus may contribute to maintaining complex floral phenotypes despite gene flow. More generally, our observation that species-diagnostic SNPs are widely dispersed across the genome indicates that the complex adaptation represented by these SNPs can evolve and be maintained in the absence of tight linkage or pervasive pleiotropy.

In some systems, sets of floral syndrome QTLs are tied up together in regions of low recombination. For example, floral adaptation in *M*. *lewisii* versus *M*. *cardinalis* maps to inversions that suppress recombination among traits in hybrids [66], and floral syndromes in *Petunia* are specified by a suite of loci in very tight linkage that efficiently lock together adaptive combinations of traits [61]. By contrast, in the *P*. *barbatus* x *P*. *neomexicanus* cross, floral trait QTLs mostly map to different regions of the genome that should experience recombination. The underlying alleles would therefore become shuffled in second generation or later hybrids between bee- and hummingbird-adapted species. However, in our study, the traits under divergent selection are predicted to cause assortative mating, which may help maintain trait combinations. Overall, this genomic arrangement not only facilitates the discovery of the genetic basis for adaptive complex trait divergence, but also provides a novel opportunity to explore how multi-locus traits can be maintained by selection in the face of gene flow.

### Surprisingly small genomic footprint and lack of extended regions of elevated genomic differentiation

The genomic landscape of polymorphism and genetic divergence should reflect intrinsic genomic features, such as the density of functional sequences and recombination rate. We recovered correlations between polymorphism and intrinsic genomic features that match the predicted effects of linked selection. The observed negative relationship between levels of gene density and genetic polymorphism likely reflects stronger purifying selection acting on functional variation in gene-rich regions relative to gene-poor regions [69–72]. The observed positive relationship between recombination rate and genetic diversity likely reflects weaker effects of background selection in regions of high recombination [73,74]. These patterns are nearly universal in population genomic studies [74–76]. Genome-wide, levels of within-population genetic polymorphism are positively correlated with between-population genetic divergence. Such a pattern may arise from shared segregating variation between populations as well as shared genomic features that shape patterns of variation both within and between populations. For example, greater purifying selection in gene-rich regions results in reduced genetic diversity within populations as well as reduced divergence between populations (e.g., [72,77]).

Loci underlying reproductive isolating barriers or locally adaptive differences that distinguish taxa have been hypothesized to generate peaks or “islands” of differentiation between taxa connected by gene flow (e.g., [17,78,79]). While some theoretical studies suggest this hypothesis might not be realistic (e.g., [13,71]), the controversy has not been resolved. Empirical investigations are required. We examined whether the outlier regions corresponding to the 3 major-effect floral QTLs for key floral traits that distinguish *P*. *barbatus* from its bee-adapted relatives (flower color, nectar volume, and flower width) are detected as islands of elevated divergence. We found that these major floral syndrome QTLs—which we expect help to maintain species boundaries—do not produce large regions of differentiation. Instead, divergence between floral syndromes seems to involve fine-grained SNP differences in high recombination regions without an extended genomic signature. This finding contrasts with studies in a variety of organisms that find complex differentiation maps to discrete regions of elevated genetic differentiation between taxa (e.g., [17,80–85]), including studies in monkeyflower systems that have found that floral syndrome differences are found in extended regions of elevated divergence [77,86]. Our results suggest that complex adaptation can involve strongly differentiated loci in high recombination regions without necessarily creating surrounding islands of divergence, in accordance with other studies (reviewed by [87]).

### A proposed evolutionary scenario

Our findings present a conundrum. The species can be immediately distinguished in the field by a constellation of traits, yet the vast majority of genome-wide variation is unpredictive of species identity. At the whole genome level, the entire complex resembles a single species. It is perplexing to consider how genetic associations with species differences appear so highly localized, especially considering that observations of hybrids are quite rare. The observed data strongly suggest that much ancestral variation remains segregating throughout the species complex, which is plausible given the recency of divergence between these species [88].

It is worth considering whether invoking gene flow is necessary to explain the observed data. There are 2 difficulties with a scenario without gene flow (where the species have remained fully isolated following divergence). First, the approximately 20 independent selective sweeps of derived hummingbird alleles that generate species diagnostic regions are highly localized and do not exhibit an extended molecular signature. Under a full isolation scenario, localized signatures might suggest that the sweeps occurred over a long period of time, either slowly or with strong recombination near selected loci. Yet, there is minimal divergence in the rest of the genome. Second, we observe a pattern of interspecific isolation-by-distance. Hummingbird-adapted populations are more similar to nearby bee-adapted populations than to geographically distant hummingbird-adapted populations (Fig 1C). This is a basic prediction of local gene flow. In principle, such a pattern could arise under a full isolation scenario if each species independently experiences a form of geographic adaptation that causes parallel genome-wide divergence. However, independent adaptation by different species to similar regions is unlikely to cause parallel change at all SNPs (which generates the genome-wide pattern in relatedness). Gene flow, even if rare, provides the clearest explanation for interspecific isolation-by-distance and also explains how the original sweeps may have a highly localized signature. Important questions remain concerning how much ancestral variation is still sorting relative to that being shared through introgression between species. It is unclear with the current data how much ongoing gene flow is occurring, but it seems to have occurred at least in the recent past. More complete sequencing in future studies will enable estimation of divergence times between adaptive alleles.

## Conclusions

The *barbatus*–*neomexicanus–virgatus* species complex is an attractive group of species to examine the origin and maintenance of complex phenotypic divergence. Overall, our data suggest that patterns of genetic differentiation between species are rapidly homogenized by gene flow, except for loci that underpin species differences. This highly localized genomic pattern contrasts with systems where islands of divergence have been found. Strong selection combined with assortative mating conferred by distinct pollinator preferences in the face of at least low levels of recent gene flow may explain these observations. Overall, the study system is ideal for association mapping, even using structured population sampling, potentially enabling us to delimit gene regions responsible for species differences with higher resolution than can be achieved by QTL mapping approaches. The shared genetic variation between species may reflect recent gene flow in secondary contact following range expansion, segregating genetic variation that predates speciation events and that is still sorting, or more complex scenarios of genetic exchange including introgression of floral syndrome traits into new genetic backgrounds.

## Materials and methods

### Ethics statement

Sampling occurred under US Forest Service permits Wessinger_R4_2016, R30 R0 262, Wessinger2016, 551 010851, R311, 2018–04, and 2021-0231-RR01.

### Genome assembly

We used a combination of short-read Illumina, long-read PacBio, and Bionano sequence data to assemble the genome of a *P*. *barbatus* individual originally collected in Gunnison County, Colorado. A voucher for this collection is deposited as CAW14 in the McGregor Herbarium of the University of Kansas Biodiversity Institute and Natural History Museum. We extracted high molecular weight DNA from flash-frozen leaf tissue using a modified CTAB protocol [89]. The Duke University Genome Sequencing core prepared Illumina libraries and performed sequencing of 2 lanes of 100 base pair (bp) paired end and 1 lane of 100 bp mate-paired sequencing (insert size of 4 kb) on the Illumina HiSeq 2500 platform. The Duke University core also prepared and sequenced libraries on the PacBio RSII platform (90 SMRT cells). The McDonnell Genome Institute (MGI) at the Washington University School of Medicine in St. Louis prepared and sequenced libraries on the PacBio Sequel II CLR platform (4 SMRT cells). The MGI also prepared Bionano libraries and conducted Bionano optical mapping using a single flowcell of a Saphyr chip, collecting images of molecules greater than 150 kb and with a minimum of nine DLE-1 enzyme labels.

An initial scaffold-level assembly of Illumina and PacBio data was constructed using the *FALCON* assembly pipeline [90], with default parameters suggested for large genome data. Errors in the resulting raw contig sequences were corrected using *Arrow* [91]. The error-corrected assembly contigs were polished to reduce residual indel errors using *Pilon* [92]. This assembly yielded 5,110 contigs, for a total assembly size of 820 Mbp (N50 = 347 kb). Bionano data were assembled using the *Bionano Solve* pipeline (Bionano Genomics) into 105 genome contigs with a total length of 1,001.5 Mbp (N50 = 16.16 Mbp). Hybrid scaffolding of the *FALCON* assembly using the Bionano assembly was performed by the MGI.

Given that we sequenced an outbred *P*. *barbatus* individual, we expected that our assembly included redundancy due to split haplotypes. We used *Haplomerger2* [93] to collapse such regions, assuming 0.5% divergence between diploid alleles, which effectively reduced redundancy while retaining *BUSCO* loci. We then used *sealer* [94] to fill N-gaps. These efforts yielded a high-quality draft assembly with 28 scaffolds and total length of 599 Mbp. We used the *BUSCO* pipeline [95] with the eudicots db10 database to assess the degree to which this assembly is complete, based on the proportion of 2,326 single-copy plant genes included in the assembly (S5 Table). We found 95% of *BUSCO* genes are complete, 9.2% are duplicated, and 4.2% are missing.

### Linkage map construction

We previously generated a linkage map and mapped floral trait QTLs using a small mapping population of 96 F2 individuals derived from a cross between *P*. *barbatus* and the related bee-adapted species *P*. *neomexicanus* [33]. Here, we used genotype data from an expanded collection of 531 F2 individuals to order and orient the 28 scaffolds into 8 chromosomes. We propagated F2 progeny at Duke University and the University of Kansas. We used MSG [32] to genotype F2 individuals and each parent individual. Briefly, we extracted DNA from fresh leaf tissue using a modified CTAB protocol and prepared MSG libraries using the restriction enzyme *AseI* to generate genotype data for each individual as previously described [33]. Libraries were sequenced on Illumina HiSeq or NextSeq platforms using single-end 150 bp reads lanes at the University of Kansas Genome Sequencing Core.

We demultiplexed and quality filtered raw sequencing reads using steps 1 and 2 of the *ipyrad* pipeline [96]. Specifically, reads were trimmed to a minimum length of 75 bp if they contained adapter sequence and were discarded if more than 5 bases had a phred quality score less than 20. We mapped filtered reads to the *P*. *barbatus* genome assembly using *bwa mem* [97], generating a mapping file for each individual. We used the UnifiedGenotyper algorithm of *GATK* [98] to identify SNPs within the sample of F2 individuals and the parent individuals, yielding a raw set of 1,666,458 SNPs. We filtered this dataset to retain only SNPs with a phred-score mapping quality score greater than 30 and that represent fixed differences between the 2 parent species (*P*. *barbatus* and *P*. *neomexicanus* parent samples are homozygous for alternate alleles), resulting in 77,773 SNPs.

Heterozygous sites tend to be underdetected in MSG datasets because the PCR amplification step during MSG library preparation can cause 1 allele to preferentially amplify by chance, resulting in apparent homozygosity. This bias against heterozygous calls is relevant for linkage map construction because sites with minimal deviation from Hardy–Weinberg equilibrium (HWE) genotype proportions are most useful. We confirmed that heterozygous sites are underdetected in our dataset—in fact individuals with lower sequence coverage across SNPs (perhaps lower quality DNA samples) tended to have a low overall proportion of heterozygous genotype calls (S7 Fig). We used 272 individuals with highest overall proportions of heterozygous genotype calls to generate a high-quality linkage map for orienting scaffolds. We then pared down our dataset to include only those SNPs present in at least 100 individuals, keeping a single SNP per MSG locus (MSG locus defined here as an individual 150 bp region sequenced by the MSG approach), with allele frequency across F2s between 0.4 and 0.6, and showing minimal deviation from HWE genotype proportions (*P* > 0.01).

We constructed a linkage map using *Lep-MAP3* [99] with imputation of missing genotypes. We assigned markers to linkage groups using an LOD threshold of 39, which separated markers into 8 linkage groups, each with at least 200 markers. We then used *Lep-MAP3* to order markers along each linkage group. Seven of the 28 scaffolds were clearly chimeric, made up of segments that map to different linkage groups. We suspected that these chimeric scaffolds were misjoined during the *haplomerger2* step. We used the genome alignment algorithm *satsuma* [100] to align our post-haplomerger genome assembly to the pre-haplomerger assembly. This allowed us to identify breakpoints in the chimeric scaffolds that represent spurious assembly of different molecules. See S1 Text for details. Three of the 28 scaffolds could not be tied to the linkage map: scaffold 23 (2,922,492 bp) that predominantly contains uncalled bases (Ns), scaffold 27 (31,112 bp), and scaffold 28 (21,378 bp). We reordered scaffolds and scaffold segments based on linkage relationships to obtain pseudochromosomes (hereafter, “chromosomes”) (S6 Table). We estimated a final genetic map for our chromosome-level assembly using *Lep-MAP3* (S8 Fig). We numbered and oriented chromosomes according to a previous linkage map for this genetic cross [33]—we established homology by aligning MSG sequences from our previous analysis to the genome assembly using *bwa mem*.

### Reestimation of floral syndrome QTLs

We reestimated floral trait QTLs analyzed in our previous QTL study [33] using the updated genetic map, allowing us to define QTL intervals based on their physical location in the genome. These traits include flower color, nectar production, and morphological traits that define pollination syndrome in *Penstemon*. Focusing on 133 F2 individuals with both phenotypic and MSG data, we generated an SNP dataset that included biallelic SNPs representing fixed differences between the parents, paring this down to SNPs found in at least 50 of the 133 F2 individuals, 1 per locus, with allele frequency within the focal set of F2s between 0.3 and 0.7, and with minimal deviation from HWE proportions (*P* > 0.0001). We ordered the resulting set of 7,396 SNPs based on their genomic coordinates and estimated genetic distances between markers.

We used *R/qtl2* [101] for QTL analysis. First, we removed redundant markers, leaving 974 recombination-informative markers. We inserted pseudomarkers every 1 cM and used the Haley–Knott method of scan1 function of *R/qtl2* to identify QTL positions and assessed significance using trait-specific LOD thresholds with 10,000 permutations of the genotype-phenotype matrix. For each significant QTL, we recorded peak position in centimorgans (cM) and confidence intervals representing a drop in LOD score of 1.5. We found the physical positions of QTL peaks and 1.5 LOD intervals using linear interpolation of physical positions of adjacent markers.

### Transcriptome assembly and genome annotation

We extracted RNA from flash frozen root, stem, and floral tissues of *P*. *barbatus* using the RNeasy Plant Mini kit (Qiagen) and used the NEBNext Ultra II Directional RNA Library prep kit for Illumina (NEB) with the NEBNext Poly(A) mRNA Magnetic Isolation Module (NEB) for Illumina sequencing library preparation. RNA was sequenced using 100 bp paired-end reads on the Illumina NovaSeq 6000 platform at the University of Kansas Medical Center Genome Sequencing Core. We quality filtered and trimmed RNA reads using *fastp* [102] and assembled a genome-guided transcriptome using *Trinity* [103,104]. We identified and classified repeat sequences using *RepeatModeler* [105]. Then, we used *MAKER* [106] to annotate genes using the genome-guided transcriptome assembly and protein evidence from the *Arabidopsis thaliana* UniProt database (downloaded September 7, 2021; The UniProt Consortium 2019). To predict gene models, we used *SNAP* [107] trained with *P*. *barbatus* transcriptome data, and *AUGUSTUS* [108] trained with *Solanum lycopersicum* (tomato) gene models. We performed functional annotation using the tomato UniProt database (downloaded January 28, 2022) with *BLAST+* [109] and found protein domains using *InterProScan* [110,111].

### Relationship between gene density and recombination rate

For non-overlapping genomic windows (500 kb and 100 kb), we tallied the number of annotated genes per interval where we assigned genes to windows based on their midpoint bp position. We found the number of coding sites per window based on annotated gene models with the aid of the *BSgenome Bioconductor* R package [112]. We estimated approximate recombination rate per window by first estimating cM positions of the bp positions delimiting each window and then extrapolating an approximate recombination rate as cM per Mbp.

### Population genomic sampling of *P*. *barbatus*, *P*. *neomexicanus*, and *P*. *virgatus* individuals

We sampled leaf material of individuals from *P*. *barbatus* populations, as well as related bee-adapted species *P*. *neomexicanus*, and *P*. *virgatus* populations, in Arizona, Colorado, and New Mexico during the summers of 2016, 2017, 2018, and 2021 for genomic analysis. We sampled 8 individuals per population for each of 15 populations of *P*. *barbatus*, 3 populations of *P*. *neomexicanus*, and 8 populations of *P*. *virgatus* (S1 Table). Based on genetic relationships among samples discovered during genetic analyses, we determined that one of the samples labeled as population CAW62 was incorrectly labeled and clearly belongs to population CAW63. This resulted in 7 individuals for CAW62 population and 9 for CAW63. We additionally included individuals from 3 outgroup populations: *P*. *pseudoputus* (8 individuals), *P*. *putus* (8 individuals), and *P*. *strictus* (5 individuals). Sampling occurred under US Forest Service permits Wessinger_R4_2016, R30 R0 262, Wessinger2016, 010851, R311, 2018–04, and 2021-0231-RR01.

We extracted DNA from each individual and generated libraries for MSG following the approach used previously [113]. Briefly, we digested DNA with the frequently cutting restriction enzyme *Mse*I and selected fragments 250 to 450 bp in length. We sequenced the MSG libraries using Illumina 150 bp single-end read sequencing on the HiSeq or NextSeq platforms at the University of Kansas Genomics Core facility using. We demultiplexed and quality-filtered sequencing reads using steps 1 and 2 of the *ipyrad* pipeline and aligned filtered reads to our reference genome using *bwa mem*. We then called genotypes using the UnifiedGenotyper method of *GATK* and emitting all confident sites (variant and invariant). We next filtered out sites with a mapping quality score <30 or more than 2 alleles, ignoring indels. We calculated the number of reads and average read depth per MSG locus and removed loci with a total read depth across individuals that was greater than the 95% quantile of total read depth across loci (466 reads).

### Pairwise nucleotide distance and *F*_*ST*_ calculations

To examine patterns of genetic divergence between *P*. *barbatus* populations and populations of the related bee-adapted species, we calculated average pairwise nucleotide distances between all individuals in our study using the following method. First, we filtered our population genomic dataset to include sites found in at least 2 individuals in our study. For each pair of individuals, we tallied the number of mismatched genotypes, where individuals showing alternative homozygous genotype calls accrued 1 mismatch and heterozygous genotypes accrued one half mismatch. We divided the number of mismatches by the total number of sites for which both individuals had data. Even though heterozygote genotype calls are likely under-called in our study due to shallow coverage, this does not bias our genetic distance calculations.

We refer to average pairwise genetic distance within populations as *π* and average pairwise genetic distance between populations as *d*_*XY*_; however, these calculations done in identical manner. Based on genome-wide averages of pairwise genetic distance matrix, we generated a distance-based (neighbor-joining) tree using the R package *ape* [114] and performed multidimensional scaling on our distance matrix using the R package *stats*. We also performed pairwise distance calculations separately for genomic windows (500 kb and 100 kb) in order to examine the topography of genetic divergence across the genome. We calculated these statistics for the original population genetic dataset as well as for a dataset that includes only 4-fold degenerate coding sites.

To examine relative differentiation between populations, we estimated *F*_*ST*_ for each pair of populations as:

$F_{ST}=\frac{\pi_{pool}-\pi_{wi}}{\pi_{pool}}$, where *π*_*wi*_ is average pairwise distance between individuals sampled from the same population and *π*_*pool*_ is average pairwise distance between individuals sampled without respect to population [115,116].

### SNP-level test for an association between genotype and pollinator adaptation

We filtered our population genomic dataset to include biallelic SNPs that are polymorphic within the set of *P*. *barbatus*, *P*. *neomexicanus*, and *P*. *virgatus* samples. We first tallied the number of sites that are fixed in *P*. *barbatus* relative to the 2 bee-adapted species, as well as tallied sites segregating within a single species or segregating across multiple species. Of 12,869,644 filtered biallelic SNPs in our population genomic dataset, only 1.5% appear fully fixed differences between *P*. *barbatus* relative to the bee-adapted species. Many of these fixed SNPs have low coverage across samples suggesting they may spuriously appear fixed due to a tiny sample size. The remaining set of SNPs in our dataset were segregating in both hummingbird- and bee-adapted individuals (36.4%), segregating in *P*. *barbatus* only (36.1%), or segregating within the sampled bee-adapted individuals only (25.9%).

To relax the requirement for fixed differences while still identifying SNPs strongly differentiated between *P*. *barbatus* versus the bee-adapted species, we conducted an SNP-by-SNP test for an association between pollination syndrome (e.g., being *P*. *barbatus* versus bee-adapted) using a likelihood-based approach that accounts for genotype uncertainty. To gain necessary power for this approach, we first filtered our biallelic variant dataset to include sites with genotype data in at least 40 hummingbird-adapted (*P*. *barbatus*) individuals and at least 40 bee-adapted (*P*. *neomexicanus* or *P*. *virgatus*) individuals, and with overall allele frequency between 0.2 and 0.8, yielding a set of 194,672 SNPs.

At each SNP for each individual, the *GATK* variant calling algorithm counts the number of reads matching the reference base (“ref allele”) and the number of reads carrying the alternative base (“alt allele”). Based on these read counts, *GATK* assigns posterior genotype likelihoods for each of the 3 possible diploid genotypes, where *L*_*g*_ is the likelihood of the observed read data if the true genotype is *g*. For a given SNP, we summed the log-likelihood of the observed set of genotypic and floral syndrome data across *n* individuals.

$$\ln\;L=\sum_{j=1}^{n}\ln\left(P[{data}_{j}]\right)$$

where *P*[data_*j*_] is the probability of the observed genomic and phenotypic data for the *j*’th individual. This probability can be written as the sum of conditional probabilities of the observed floral syndrome phenotype *Z* in a given genotype, multiplied by the genotype probability:

$$P[{data}_{j}]=P[Z|RR]P[RR]+P[Z|RA]P[RA]+P[Z|AA]P[AA].$$

Here, RR denotes the genotype that is homozygous for the ref allele, RA is heterozygous, and AA is homozygous for the alt allele. We found genotype probabilities by multiplying posterior genotype likelihoods emitted from GATK by the prior genotype probabilities based on *q*, the overall frequency of the reference base within the set of individuals [113,117].


$$P[RR]=L_{RR}q^{2}$$

$$P[RA]=L_{RA}2q(1-q)$$

$$P[AA]={L_{AA}(1-q)}^{2}.$$

We consider floral syndrome as a binary trait, with *P*. *barbatus* individuals having hummingbird syndrome flowers (*Z* = 1) and *P*. *neomexicanus* and *P*. *virgatus* individuals having bee syndrome flowers (*Z* = 0). Let *p* equal the probability that a given individual has hummingbird syndrome flowers:

**Table:** 

Floral syndrome	*P*[*Z*|RR]	*P*[*Z*|RA]	*P*[*Z*|AA]
Hummingbird	*p*_*RR*_	*p*_*RA*_	*p*_*AA*_
Bee	1 –*p*_*RR*_	1 –*p*_*RA*_	1 –*p*_*AA*_

We fit a model (*H*_*1*_) where the probability of being hummingbird adapted depends on genotype (*p*_*RR*_, *p*_*RA*_, and *p*_*AA*_ are free to vary) and compared this to a null model (*H*_*0*_) where the probability of being hummingbird-adapted flowers does not depend on genotype (*p*_*RR*_ = *p*_*RA*_ = *p*_*AA*_). For each site, we compared the likelihood of *H*_*1*_ to *H*_*0*_ using the LRT statistic and tested significance using the chi-square distribution with 2 degrees of freedom. We performed calculations on the filtered dataset using a custom Python script that maximizes likelihoods with Broyden–Fletcher–Goldfarb–Shanno (BFGS) bounded optimization implemented in SciPy (www.scipy.org).

To address whether the coincidence of SNP association with outlier status (LRT value >100 and allele frequency difference between species >0.9) is greater than expected under random chance, we randomly permuted the positions of SNPs in our dataset 10,000×. For each permutation, we recorded the number of SNPs falling within 100 kb of a QTL peak LOD position and compared this distribution to the observed number of SNPs meeting this threshold.

### Dryad DOI

10.5061/dryad.xpnvx0kmp [118]

## Supporting information

S1 Text Identification of chimeric scaffold breakpoints. (PDF)

S1 Table Population sampling of *P*. *barbatus*, *P*. *neomexicanus*, and *P*. *virgatus*. (XLSX)

S2 Table Between-population distance (*d*_*XY*_) and differentiation (*F*_*ST*_) for pairwise population contrasts. Reported are median values and ranges.
(XLSX)

S3 Table Attributes of the 63 species-diagnostic SNPs. *q*_*bar*_: frequency of the reference allele within the *P*. *barbatus* samples, *q*_*bee*_: frequency of the reference allele within the bee-adapted samples.
(XLSX)

S4 Table Floral trait QTLs, grouped by trait. (XLSX)

S5 Table BUSCO information for the final genome assembly, using the eudicots db10 dataset. (XLSX)

S6 Table Distribution of chromosome lengths in *P*. *barbatus* genome. (XLSX)

S1 Fig Average pairwise genetic differentiation (*F*_*ST*_) between populations. (A) Pairwise differentiation as a function of geographic distance. (B) Pairwise population differentiation summarized by contrast type. Red: pairwise contrasts between *P*. *barbatus* populations, green: pairwise contrasts between *P*. *barbatus* and bee-adapted populations. *b*: *P*. *barbatus*, *n*: *P*. *neomexicanus*, *v*: *P*. *virgatus*. The data underlying this figure can be found in 10.5061/dryad.xpnvx0kmp.
(PDF)

S2 Fig The relationship between the likelihood ratio test (LRT) statistic value for SNP associations and the absolute difference in allele frequency between *P*. *barbatus* vs. bee-adapted populations. The thresholds for identifying species-diagnostic regions (allele frequency difference >0.9 and LRT >100) are shown with gray dotted lines and the species-diagnostic SNPs are highlighted in red. The data underlying this figure can be found in 10.5061/dryad.xpnvx0kmp.
(PDF)

S3 Fig Locations of species diagnostic sites plotted above QTL LOD traces. Top panel in each plot shows SNP associations with species identity according to the strength of the likelihood ratio test statistic (LRT). Points highlighted in red are outlier SNPs (difference in allele frequency >0.9 and LRT >100). QTL traces are plotted according to LOD value, dotted line indicates peak LOD position. The data underlying this figure can be found in 10.5061/dryad.xpnvx0kmp.
(PDF)

S4 Fig Results of permuting SNP positions relative to QTL peak LOD positions. We randomly permuted the positions of SNPs in our population genomic dataset 10,000 times. For each permutation, we recorded the number of SNPs falling within 100 kb of a QTL peak LOD position and compared this to the number of SNPs in the observed dataset (8). The data underlying this figure can be found in 10.5061/dryad.xpnvx0kmp.
(PDF)

S5 Fig Patterns of recombination rate, gene density, and pairwise genetic distance between samples in 500 kb genomic windows. (A) Genetic distance in centimorgans (cM) as a function of physical distance in Mbp (slope reflects rate of recombination). (B) Histogram showing count of genes per window. (C) Genetic diversity (*π*) and divergence (*d*_*XY*_) within and between *P*. *barbatus* and/or bee-adapted species, plotted in moving averages of ten 500-kb windows (pink trace: average *π* within *P*. *barbatus* populations, red trace: average *d*_*XY*_ between *P*. *barbatus* populations, green trace: average *d*_*XY*_ between *P*. *barbatus* and bee-adapted populations). In all panels, colored vertical lines show peak LOD positions for floral trait QTLs identified in a genetic cross between *P*. *barbatus* and *P*. *neomexicanus*. See Fig 2 for key to QTL colors. The data underlying this figure can be found in 10.5061/dryad.xpnvx0kmp.
(PDF)

S6 Fig Relationships between genomic features and patterns of genetic variation. Relationships are shown between genomic features (recombination rate (cM/bp) and fraction of coding sites) and average within-population polymorphism (*π*), average *d*_*XY*_ between *P*. *barbatus* vs. bee-adapted populations, or average *F*_*ST*_ between *P*. *barbatus* vs. bee-adapted populations, for 4-fold degenerate sites in 500 kb genomic windows that contain data for at least 500 sites. Orange indicates windows that contain peak LOD positions for floral trait QTLs. The data underlying this figure can be found in 10.5061/dryad.xpnvx0kmp.
(PDF)

S7 Fig The positive relationship between proportion of heterozygous genotypes and the total number of SNP genotypes per individual in our F2 MSG dataset. The data underlying this figure can be found in 10.5061/dryad.xpnvx0kmp.
(PDF)

S8 Fig Plot of genetic map estimated by *Lepmap3*. The data underlying this figure can be found in 10.5061/dryad.xpnvx0kmp.
(PDF)

## Abbreviations

BFGS: Broyden–Fletcher–Goldfarb–Shanno
HWE: Hardy–Weinberg equilibrium
LOD: logarithm of odds
LRT: likelihood ratio test
MGI: McDonnell Genome Institute
MSG: multiplexed shotgun genotyping
QTL: quantitative trait locus
SNP: single nucleotide polymorphism
