# Supplementary material for: A few essential genetic loci distinguish Penstemon species with flowers adapted to pollination by bees or hummingbirds
Source: PLoS Biol. 2023 Sep 28;21(9):e3002294. doi: 10.1371/journal.pbio.3002294 (PMC10538765; doi:10.1371/journal.pbio.3002294)
Supplement: S1 Text — (PDF) [file pbio.3002294.s001.pdf]

S1 Text. Identification of chimeric scaffold breakpoints

We identified the breakpoints of chimeric scaffolds using the following approach. First, we identified the boundaries for a potential breakpoint based on the physical positions of included markers and their linkage relationships, as determined through *Lepmap3* linkage map construction. Next, we aligned the original (pre-haplomerger) scaffolds to the final (post-haplomerger) assembly to identify breaks between original scaffolds. Finally, we took the halfway position between the breaks between aligned scaffolds as the breakpoint for splitting chimeric scaffolds.

Scaffold	Interval from Lepmap3	Break found in original scaffolds	Halfway point
4	38,876,432 – 40,394,919	40,152,930 – 40,154,590	40,153,760
4	66,190,526 – 67,241,756	68,165,080 – 68,175,110	68,170,095
5	5,783,258 – 7,201,059	5,911,240 – 5,914,660	5,912,950
5	18,461,260 – 18,882,572	18,479,530 – 18,487,250	18,483,390
5	26,444,421 – 26,712,503	26,636,280 – 26,628,090	26,632,185
7	12,688,620 – 17,374,066	No break in original scaffolds	There is a large 2,229,339 bp N-gap from 12,885,401 – 15,114,740
15	14,623,416 – 14,667,851	14,723,310 – 14,749,680	14,736,495
16	13,687,759 – 14,142,018	14,020,040 – 14,026,290	14,023,165
17	6,425,389 – 7,377,148	6,045,980 – 6,051,770	6,048,875
20	3,668,937 – 3,911,598	3,948,600 – 3,961,550	3,955,075

Some of the scaffolds (or scaffold segments) were difficult to orient based on linkage relationships identified in *Lepmap3* because they are found in low recombination regions. These included the following scaffold segments: scaffold 1 (0 – 56 Mbp), scaffold 4 (40.2 – 68.2 Mbp), scaffold 4 (68.1 – 79.1 Mbp), scaffold 13 (0 – 15.3 Mbp), scaffold 16 (14.7 – 22.7 Mbp), scaffold 17 (0 – 6 Mbp), scaffold 17 (6 – 15 Mbp), scaffold 20 (3.9 – 10.6 Mbp), scaffold 24 (0 – 2.8 Mbp). To improve ordering in the face of genotype uncertainty at any given snp, we identified aggregate genotypes for sets of markers at the end of each scaffold segment and searched for informative recombination events across the segment that would help orient the scaffold segment based on aggregate genotypes at the ends of adjacent scaffolds. We generated hard genotype calls for each SNP in each individual using a custom python script and inspected the resulting table in excel. Through this effort we established orientation for all but three scaffold segments: scaffold 4 (68.1 – 79.1 Mbp), scaffold 17 (0 – 6 Mbp), and scaffold 24 (0 – 2.8 Mbp). We arbitrarily assigned orientation to these scaffolds. Based on this determined order and orientation, we produced a final fasta file of concatenated scaffold segments representing pseudochromosomes with 10,000 bp Ns as buffers between segments on a given pseudochromosome.
