# Supplementary figures and images for: A few essential genetic loci distinguish Penstemon species with flowers adapted to pollination by bees or hummingbirds
Source: PLoS Biol. 2023 Sep 28;21(9):e3002294. doi: 10.1371/journal.pbio.3002294 (PMC10538765; doi:10.1371/journal.pbio.3002294)

**A**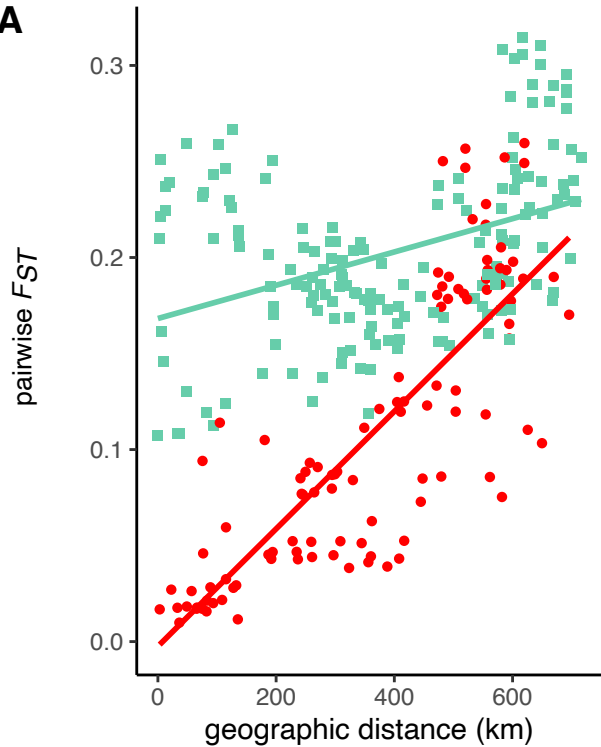**B**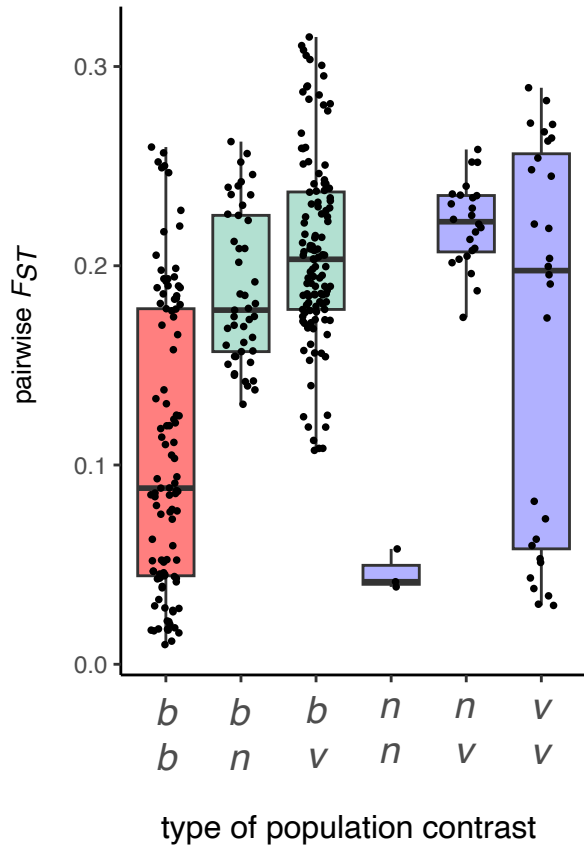

Supplement: S1 Fig — (A) Pairwise differentiation as a function of geographic distance. (B) Pairwise population differentiation summarized by contrast type. Red: pairwise contrasts between P. barbatus populations, green: pairwise contrasts between P. barbatus and bee-adapted populations. b: P. barbatus, n: P. neomexicanus, v: P. virgatus. The data underlying this figure can be found in 10.5061/dryad.xpnvx0kmp. (PDF) [file pbio.3002294.s008.pdf]

Absolute value of allele  
frequency difference

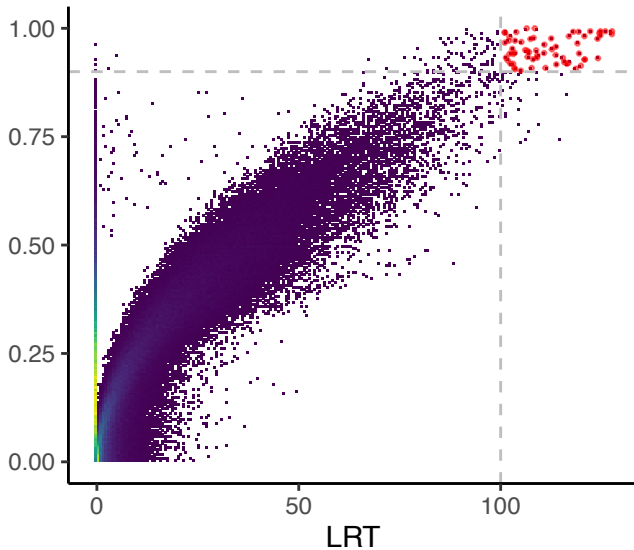

count

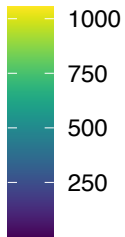

Supplement: S2 Fig — The thresholds for identifying species-diagnostic regions (allele frequency difference >0.9 and LRT >100) are shown with gray dotted lines and the species-diagnostic SNPs are highlighted in red. The data underlying this figure can be found in 10.5061/dryad.xpnvx0kmp. (PDF) [file pbio.3002294.s009.pdf]

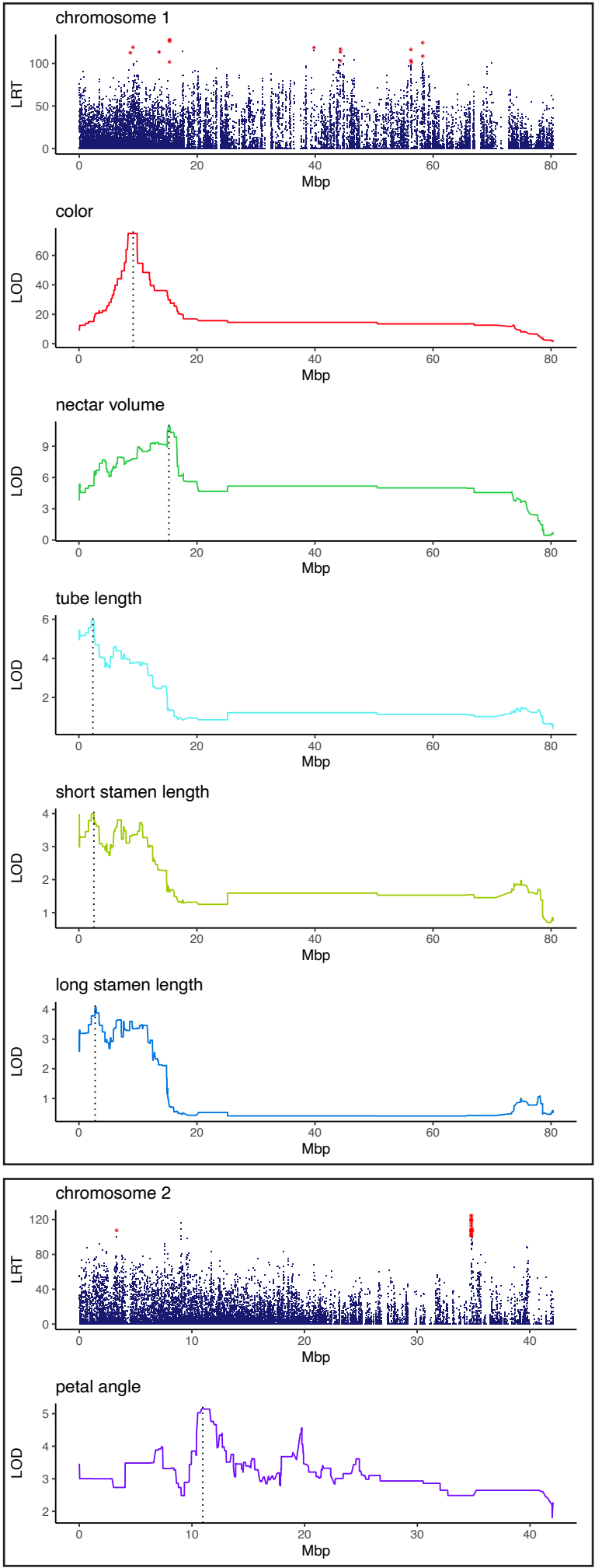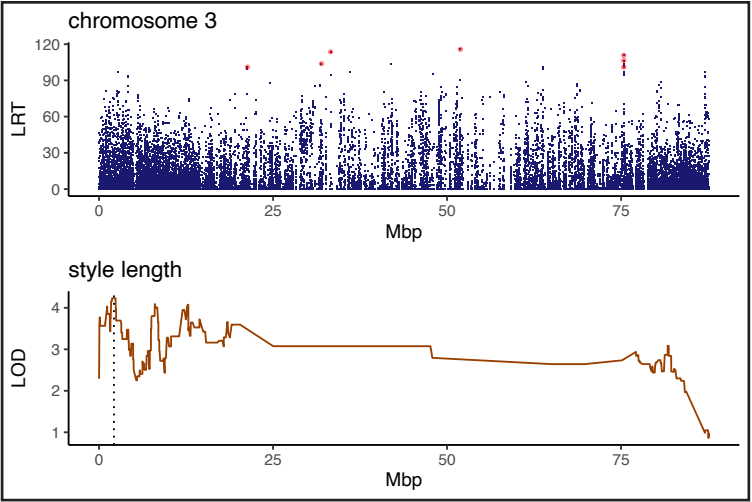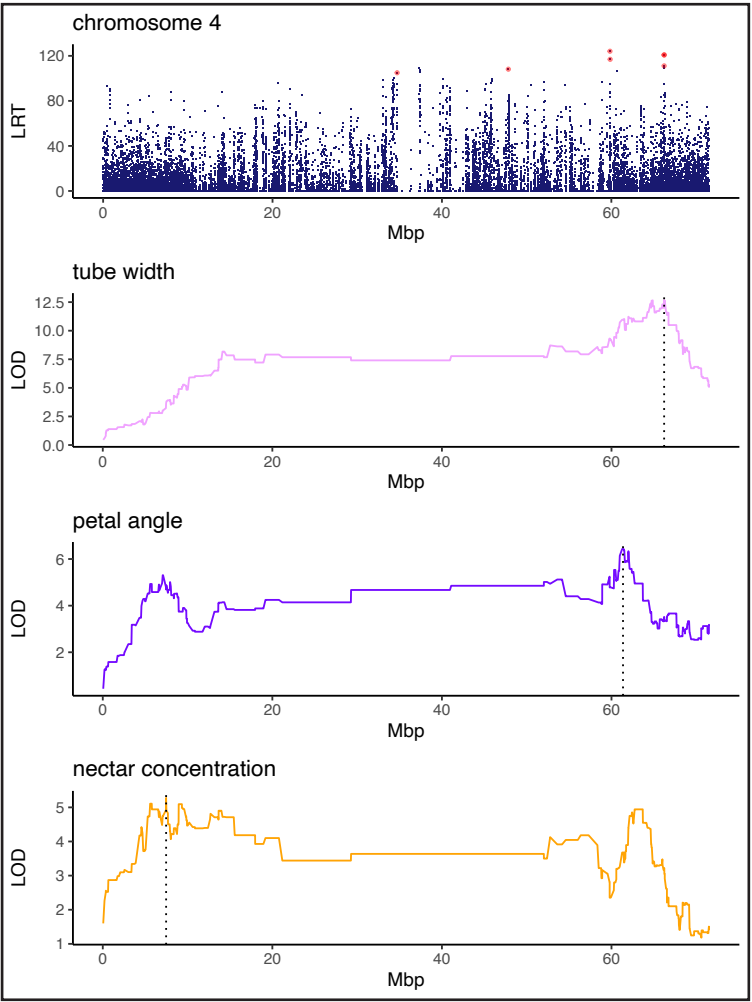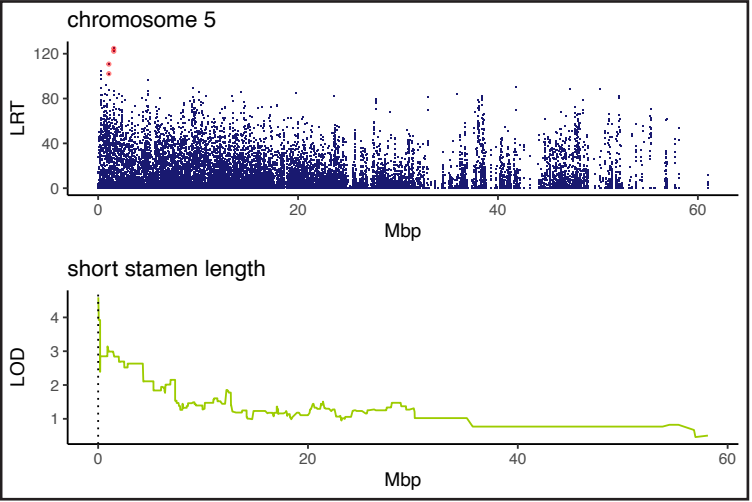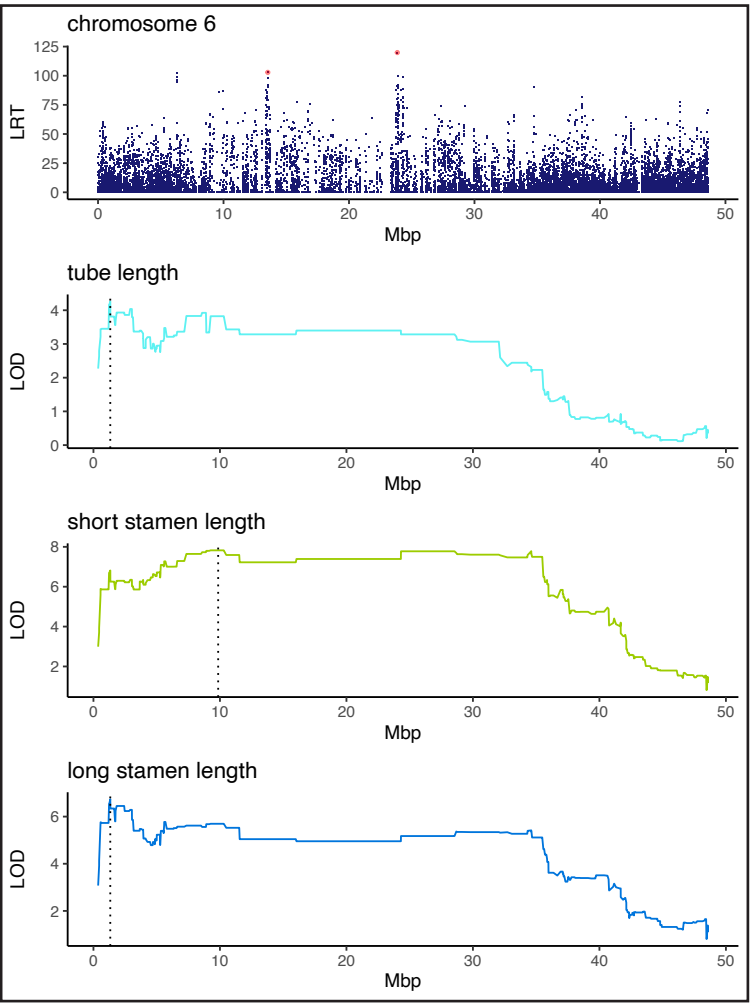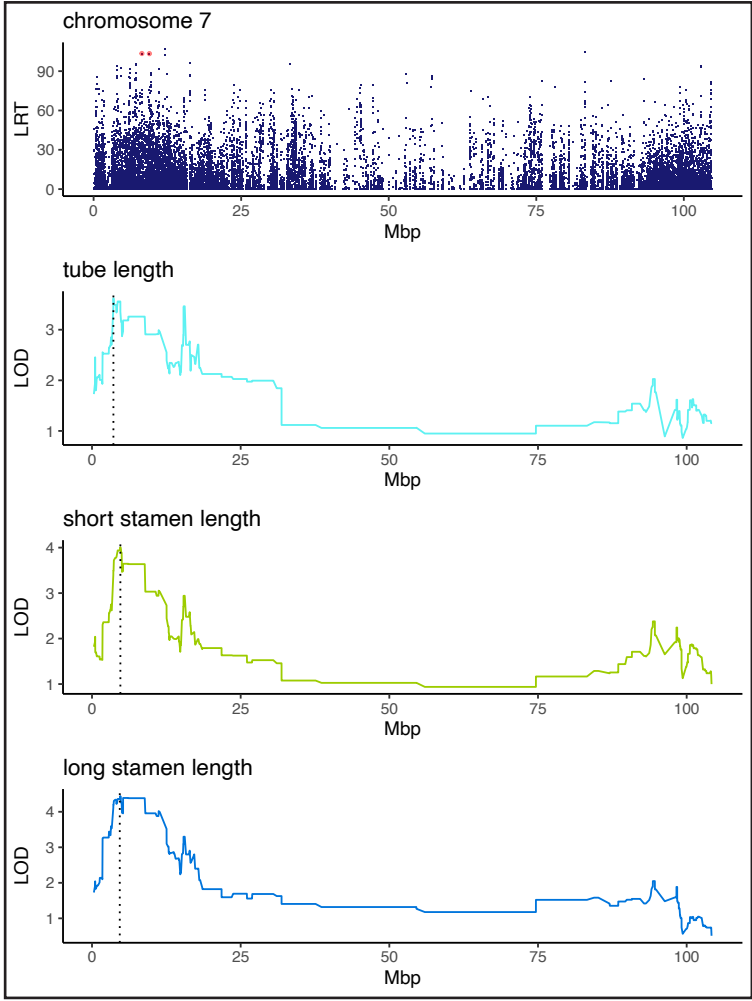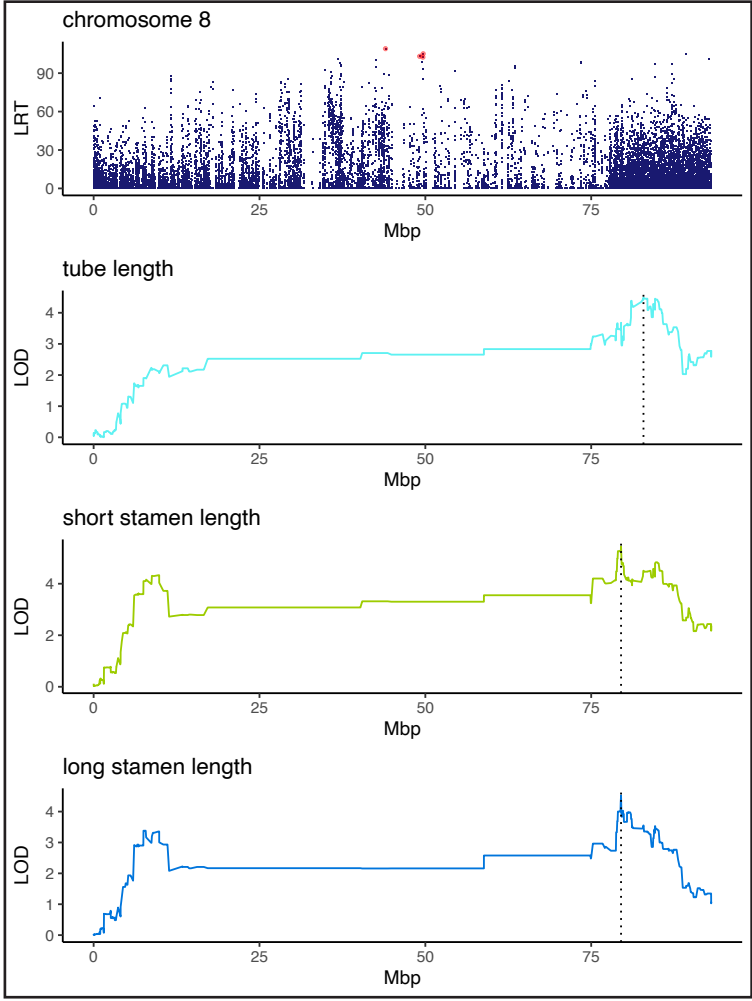

Supplement: S3 Fig — Top panel in each plot shows SNP associations with species identity according to the strength of the likelihood ratio test statistic (LRT). Points highlighted in red are outlier SNPs (difference in allele frequency >0.9 and LRT >100). QTL traces are plotted according to LOD value, dotted line indicates peak LOD position. The data underlying this figure can be found in 10.5061/dryad.xpnvx0kmp. (PDF) [file pbio.3002294.s010.pdf]

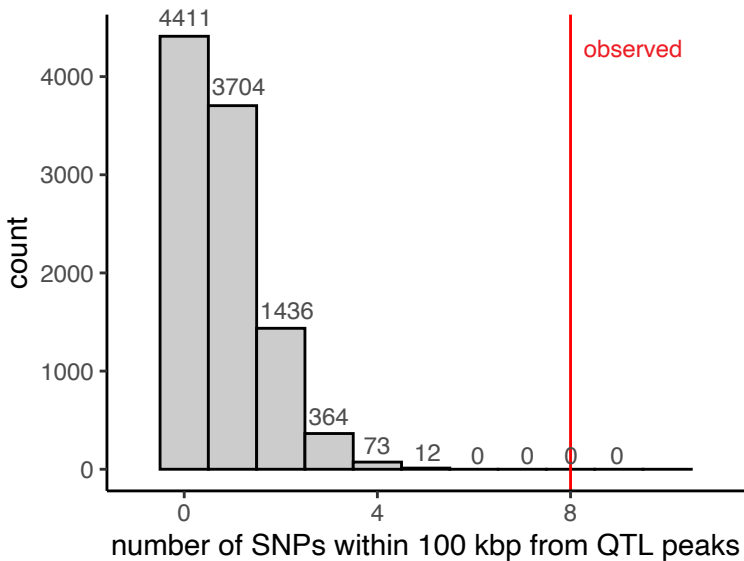

Supplement: S4 Fig — We randomly permuted the positions of SNPs in our population genomic dataset 10,000 times. For each permutation, we recorded the number of SNPs falling within 100 kb of a QTL peak LOD position and compared this to the number of SNPs in the observed dataset (8). The data underlying this figure can be found in 10.5061/dryad.xpnvx0kmp. (PDF) [file pbio.3002294.s011.pdf]

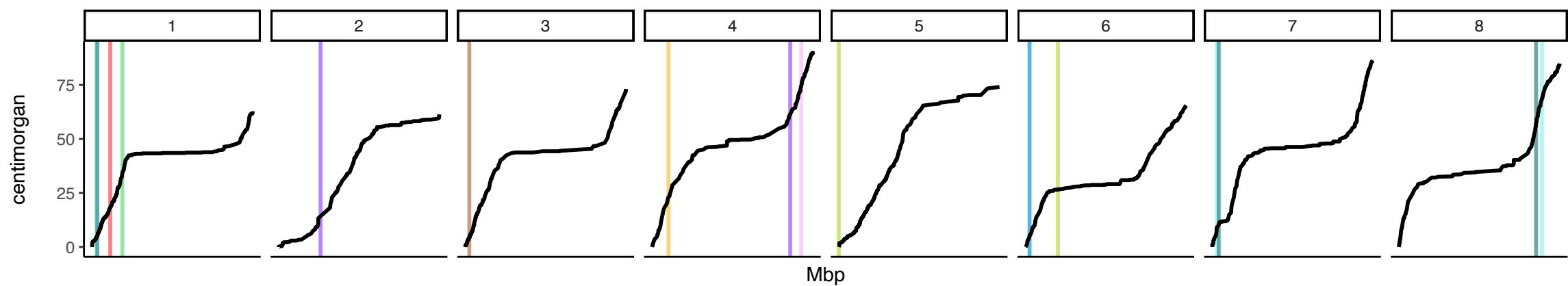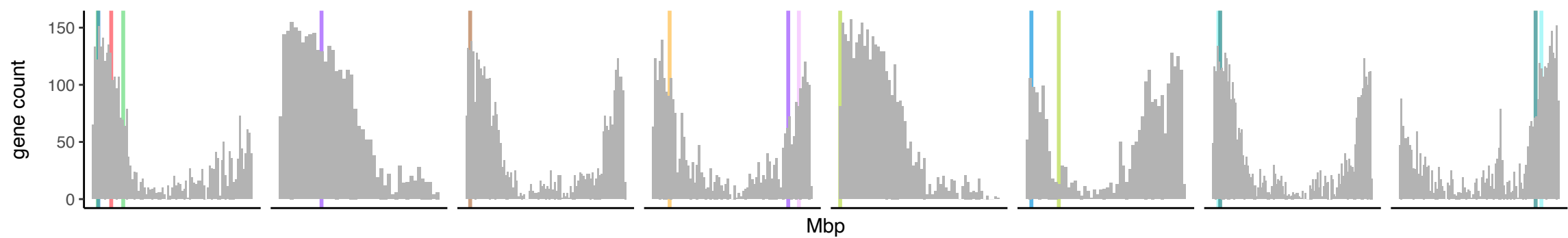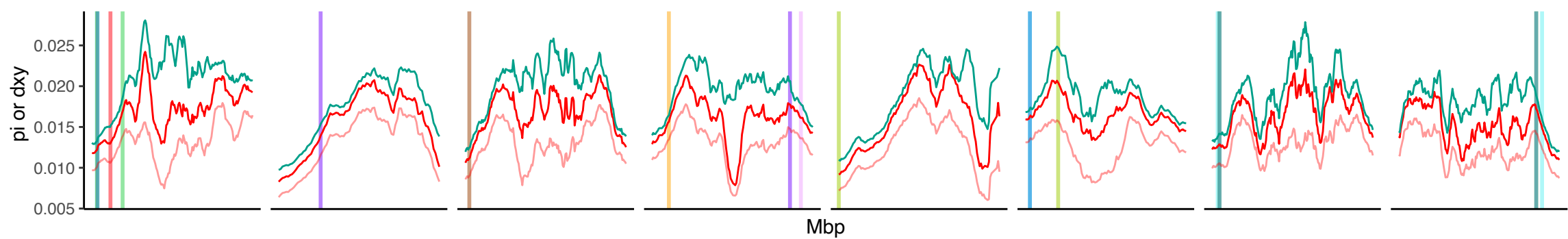

Supplement: S5 Fig — (A) Genetic distance in centimorgans (cM) as a function of physical distance in Mbp (slope reflects rate of recombination). (B) Histogram showing count of genes per window. (C) Genetic diversity (π) and divergence (dXY) within and between P. barbatus and/or bee-adapted species, plotted in moving averages of ten 500-kb windows (pink trace: average π within P. barbatus populations, red trace: average dXY between P. barbatus populations, green trace: average dXY between P. barbatus and bee-adapted populations). In all panels, colored vertical lines show peak LOD positions for floral trait QTLs identified in a genetic cross between P. barbatus and P. neomexicanus. See Fig 2 for key to QTL colors. The data underlying this figure can be found in 10.5061/dryad.xpnvx0kmp. (PDF) [file pbio.3002294.s012.pdf]

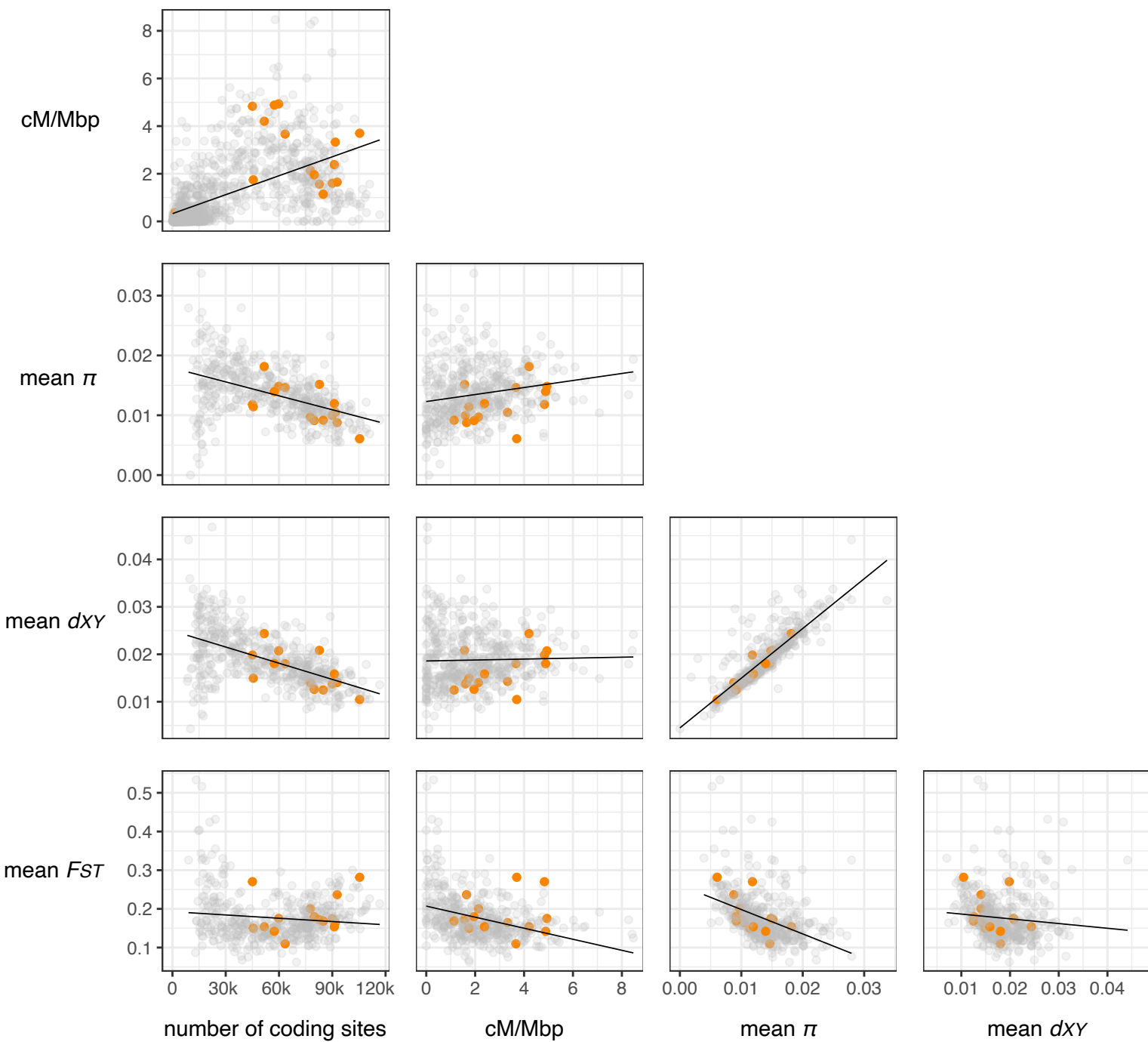

Supplement: S6 Fig — Relationships are shown between genomic features (recombination rate (cM/bp) and fraction of coding sites) and average within-population polymorphism (π), average dXY between P. barbatus vs. bee-adapted populations, or average FST between P. barbatus vs. bee-adapted populations, for 4-fold degenerate sites in 500 kb genomic windows that contain data for at least 500 sites. Orange indicates windows that contain peak LOD positions for floral trait QTLs. The data underlying this figure can be found in 10.5061/dryad.xpnvx0kmp. (PDF) [file pbio.3002294.s013.pdf]

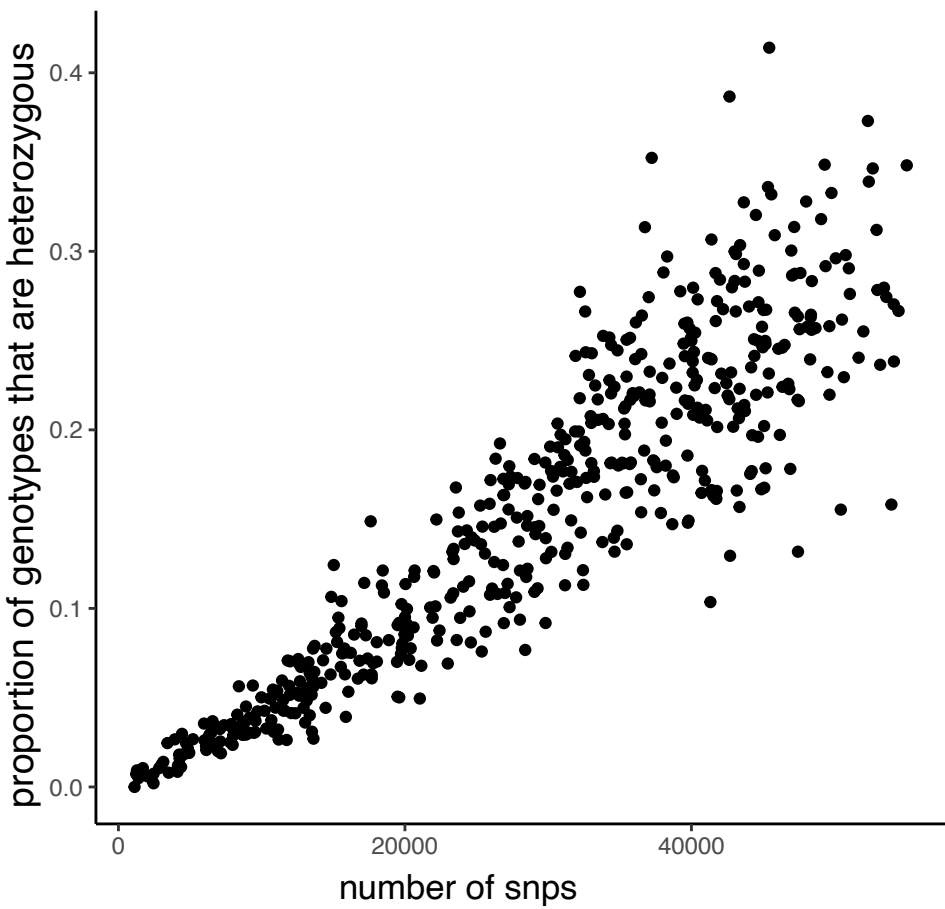

Supplement: S7 Fig — The data underlying this figure can be found in 10.5061/dryad.xpnvx0kmp. (PDF) [file pbio.3002294.s014.pdf]

# Genetic map

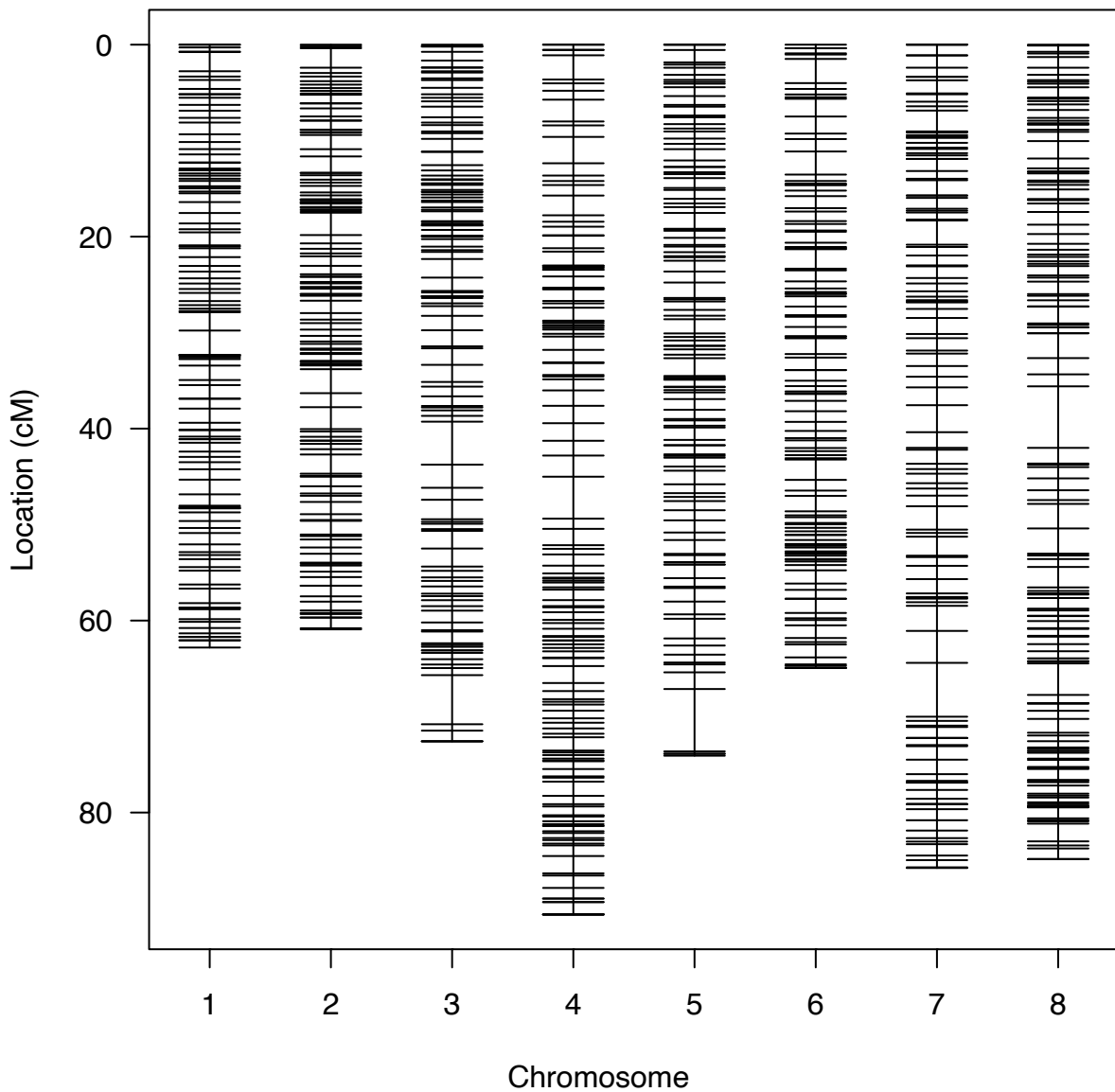

Supplement: S8 Fig — The data underlying this figure can be found in 10.5061/dryad.xpnvx0kmp. (PDF) [file pbio.3002294.s015.pdf]
